# Do Children’s Health Resources Differ According to Preschool Physical Activity Programmes and Parental Behaviour? A Mixed Methods Study

**DOI:** 10.3390/ijerph110302407

**Published:** 2014-02-26

**Authors:** Elena Sterdt, Natalie Pape, Silke Kramer, Sebastian Liersch, Michael Urban, Rolf Werning, Ulla Walter

**Affiliations:** 1 Institute for Epidemiology, Social Medicine and Health Systems Research, Hannover Medical School, Hannover 30625, Germany; E-Mails: kramer.silke@mh-hannover.de (S.K.); liersch.sebastian@mh-hannover.de (S.L.); walter.ulla@mh-hannover.de (U.W.); 2 Institute of Education for Special Needs, Leibniz Universität Hannover, Hannover 30159, Germany; E-Mails: natalie.pape@ifs.phil.uni-hannover.de (N.P.); rolf.werning@ifs.phil.uni-hannover.de (R.W.); 3 Faculty of Educational Science, Bielefeld University, Bielefeld 33501, Germany; E-Mail: m.urban@uni-bielefeld.de

**Keywords:** physical activity, social behaviour, quality of life, preschool, children, mixed methods design

## Abstract

Preschool can have positive effects on the development of a healthy lifestyle. The present study analysed to what extent different conditions, structures and behavioural models in preschool and family—children’s central social microsystems—can lead to differences in children’s health resources. Using a cross-sectional mixed methods approach, contrast analyses of “preschools with systematic physical activity programmes” *versus* “preschools without physical activity programmes” were conducted to assess the extent to which children’s physical activity, quality of life and social behaviour differ between preschools with systematic and preschools without physical activity programmes. Differences in children’s physical activity according to parental behaviour were likewise assessed. Data on child-related outcomes and parent-related factors were collected via parent questionnaires and child interviews. A qualitative focused ethnographic study was performed to obtain deeper insight into the quantitative survey data. Two hundred and twenty seven (227) children were interviewed at 21 preschools with systematic physical activity programmes, and 190 at 25 preschools without physical activity programmes. There was no significant difference in children’s physical activity levels between the two preschool types (*p* = 0.709). However, the qualitative data showed differences in the design and quality of programmes to promote children’s physical activity. Data triangulation revealed a strong influence of parental behaviour. The triangulation of methods provided comprehensive insight into the nature and extent of physical activity programmes in preschools and made it possible to capture the associations between systematic physical activity promotion and children’s health resources in a differential manner.

## 1. Introduction

Regular physical activity in childhood promotes physical, social and psychological development and energy balance [1]. Physical activity enhances gross and fine motor skills [2] as well as social skills such as peer interaction [3]. It can boost self-esteem and self-efficacy, help establish friendships and strengthen group identity [4]. Since physical activity and inactivity habits track from early childhood into adulthood [5], physical activity promotion in the preschool years represents a critical period to intervene in order to optimize health promotion [6]. 

In Germany, almost all children (93%) between the ages of three and six years and 29% of those under three years of age spend one-third of their day at preschool. There is no legal obligation to send a child to preschool. The parents decide if they want to take advantage of the available child day care services. Since August 2013, every child in Germany has a right to child care services from birth. The child day care rates that parents must pay vary greatly between different cities, municipalities and day care owners. Families with low incomes and several children pay lower fees. The high rate of use of child care facilities throughout Germany by children of preschool age attests to the high relevance of implementing health promotion activities in preschool settings.

Preschool can have a positive effect on the development of a healthy lifestyle [7,8] and support health-promoting physical activity levels in young children [6]. However, evidence suggests that preschoolers exhibit low levels of physical activity, even during preschool hours [9,10,11,12]. To date, only a few studies exist on the effects of preschool on children’s physical activity [13,14]. The available studies show consistent differences in the quality and quantity of physical activity between different preschools [12,15,16,17]. A preschool’s social and physical environment seems to be a strong predictor of preschoolers’ physical activity levels [3,15]. It is assumed that preschool-specific policies and practices, the physical design of the preschool environment and the behaviour of teachers are important factors influencing children’s physical activity [13]. Structured physical activity programmes can increase the amount and intensity of physical activity and enhance the movement skills of children [18]. 

The present study is based on social ecological models of health. Bronfenbrenner’s [19] social ecological model describes the relationship between a child’s development processes and influential environmental systems at the micro, exo and macro level [20]. Accordingly, a child’s development takes place in the context of different systems. For preschoolers, family, educators and peers are the three main system elements at the micro level. These elements should be targeted in interventions [20]. According to the biopsychosocial model of the World Health Organization [21], it is important to strengthen and promote both individual resources and systemic resources [22]. Systemic approaches to promoting resources to include the living environment through holistic consideration of structural conditions, cultural conditions, individual lifestyles and social contexts (e.g., family cohesion, social climate in settings, and interaction and communication processes).

The socialization of health behaviour occurs within the family. Parents are known to be key socializing agents of children. Their beliefs, attitudes and behaviours strongly influence their children’s health behaviour; their physical activity behaviour is considered to be one of the strongest determinants of children’s physical activity [23,24]. The social cognitive theory of Bandura [25] is the appropriate theoretical basis by which to study parent-child relationships as a correlate of physical activity while emphasizing the importance of family influence on the development of self-efficacy.

The present study aims to analyze the extent to which different conditions, structures and behaviours in preschool and family—children’s central social microsystems—can lead to differences in children's health resources (physical activity, quality of life (QoL) and cognitive skills), peer interactions and social behaviour based on socio-ecological models of health. Factors correlated with physical activity must be known in order to develop effective intervention programmes to promote physical activity in children [14,26]. The present study addresses this research deficit. 

A research design that implements and combines different methods is needed to adequately consider the complexity of the preschool setting and the multitude of biological, psychological, socio-cultural and environmental factors that determine children’s physical activity behaviour [14]. Creswell *et al*. [27] define a mixed methods study as one that “involves the collection or analysis of both quantitative and/or qualitative data in a single study in which the data are collected concurrently or sequentially, are given a priority, and involve the integration of the data at one or more stages in the process of research”. The process of combining qualitative and quantitative methods and research results can lead to an adequate understanding of the examined social processes and structures in the setting [28]. The combination of qualitative and quantitative methods can be used for (1) two-way validation of data, methods and results and (2) mutual supplementation of research results. 

Qualitative and quantitative results often appear in a different light when compared with results obtained using other methods, and such comparisons make it possible to obtain a more comprehensive picture of the research topic [28].

A baseline survey of physical activity programmes and opportunities at all preschools (n = 4,114, n = 2,415/59% returned) in Lower Saxony, the second largest and fourth most populous state in Germany, provided a starting point for the present study [29]. Data from the baseline survey formed the basis for the identification of preschools with systematic physical activity programmes (type 1) and preschools without physical activity programmes (type 2). Systematic physical activity programmes were defined as integrated, comprehensive and targeted physical activity promotion programmes which included the following five quality criteria: written physical activity policy, structured weekly physical activity offerings for all children, at least one trained physical education teacher, physical activity friendly indoor and outdoor facilities, and structured physical activity promotion in place for at least two years. 

Based on these quality criteria, it was found that 26% (n = 554) of all preschools surveyed have systematic physical activity programmes, and 3% (n = 64) lack physical activity programmes. Most (71%, n = 1,514) promote physical activity in children to some extent and were thus classified as preschools with limited physical activity programmes. See Sterdt *et al*. [29] for a detailed description of the study results.

## 2. Methods

Using a cross-sectional mixed methods approach, contrast analyses of preschools with systematic physical activity programmes (type 1) and preschools without physical activity programmes (type 2) were conducted to analyse differences in children’s physical activity behaviour, social behaviour and health resources (QoL, cognitive skills) between preschools with systematic physical activity programmes and preschools without physical activity programmes. Differences in children’s physical activity by family factors (parental socialization behaviour) were likewise investigated. 

The 20 preschools with the highest and lowest quality scores were selected from each type group based on the results of the baseline survey of preschool physical activity programmes in Lower Saxony, Germany [29]. A 2 × 2 × 2 factorial design was used for type comparison of “preschools with systematic physical activity programmes“ and “preschools without physical activity programmes” (factor 1) overall and by gender (factor 2) and socioeconomic status (low/high, factor 3). Oversampling was performed to exclude gross differences in the distribution of relevant factors such as socioeconomic status (SES) and migration background and to enable separate subgroup analyses. In other words, the number of subjects included in the study was greater than the statistically required number.

It was determined that 10 children and one of their parents had to be recruited from each of the selected preschools in order to detect effects of moderate size. Thus, 200 children and 200 parents were recruited from each preschool type group, yielding a total population of n = 400 children and n = 400 parents. 

The inclusion criteria for children were: (1) age of five to six years, (2) one participating parent and (3) attendance at a participating preschool for at least two years and at least in two-thirds day care (at least six hours of care per day). At the beginning of the study, the parents received detailed information about the research objectives and methodology and had to sign a written informed consent form allowing their child to participate. All children who met the criteria and whose parents gave their written informed consent were included in the study.

All children and parents were surveyed using largely standardised, validated instruments, which had been pre-tested at ten preschools (n = 100 children). The instruments were based on different questionnaires used in representative studies (e.g., German Health Interview and Examination Survey for Children and Adolescents—KiGGS) [30,31] to ensure a high level of data comparability. The child-related outcomes (physical activity, social behaviour, QoL) were assessed in written surveys of parents and teachers and oral interviews with the children. 

The comprehensive parental questionnaire collected information on the children’s after-school levels of moderate-to-vigorous physical activity (MVPA), in minutes per week, and on their social behaviour, QoL, and parental socialization behaviour regarding physical activity. To capture MVPA, parents were asked to indicate the number of days their child engaged in at least 60 minutes of vigorous physical active per day in a normal week, and to specify what type of activity it was. Parental socialization behaviour included the following familial factors: parental support (taking the child to sports facilities), parental attitudes towards physical activity (role of sports and physical activity in the family), and parental role model behaviour (joint parent-child physical activity).

In addition to the information provided by the parents on the children’s physical activity behaviour, pedometers were used as a tool for objective measurement of children’s physical activity levels. After obtaining parental and teacher consent for a subsample of 120 randomly selected children (60 per preschool type), the children were equipped with Omron Walking Style Pro pedometers. The children and their parents and teacher were given detailed instructions for pedometer use. The children were instructed to wear the pedometer all day for a one-week period, at all times during and after preschool, except when sleeping. 

The children’s subjective QoL was assessed using two versions of the German “Questionnaire for the Assessment of Health-related Quality of Life in Children”: one (Kiddy-KINDL-R 4-7) is used to interview children directly, and the other (KINDL R 4-7) is a written questionnaire for parents [32].

The social behaviour of the children was assessed using the Peer Problems Scale and the Prosocial Scale of the Strength and Difficulties Questionnaire (SDQ). Both the parent and teacher versions of the SDQ were used in this study. Since the study investigated the children’s social behaviour during preschool hours, the outcome variables were defined based on the information received from teachers. The SDQ scales categorize children’s social behaviour as normal, borderline or abnormal [33]. In the teacher’s version, “at least borderline” behaviour is defined as a peer problems score higher than 1.72 and a prosocial score less than 1.90. 

In addition, a qualitative focused ethnographic study was performed to deepen insights into the quantitative survey data on the associations between preschool physical activity programmes and children’s physical activity and social behaviour. The concrete physical activities of children and their social contextualization within the interaction forms and structures of activities offered at day care centres as well as a differentiated analysis of peer interactions were the main focus of observation.

The ethnographic approach made it possible to obtain insight into daily preschool routine while being directly connected to the stakeholder environment. Access to the field of study was gained through participative observation. This method makes it possible to gather data on the acceptance and utilization of physical activity options by children and other data largely lacking in the research landscape. Focused ethnography “focuses on small elements of one’s own society” [34]. The use of a focused approach was substantiated by the rather short field visits based on which daily routine at the preschools was depicted. 

Each systematic physical activity and non-physical activity preschool with the highest and lowest quality score (two each) was included in a subsample to investigate the practices of these preschools through participation in the field and to observe these children’s physical activity and social behaviour. Two researchers observed each preschool for 10 to 12 days for an observation period of at least three weeks per preschool. Field notes [35,36] were directly transferred into detailed observation logs [37]. The observations captured the specific dynamics and characteristics of the field as observed during teachers’ meetings, preschool work, and free play, *etc*. To validate the observations, ethnographic interviews were conducted with teachers and parents as well as with the children themselves.

### 2.1. Data Analysis

The analysis of quantitative data for verification of differences in physical activity and social behaviour of children between the preschool groups was primarily descriptive. Different types of variance analysis (*t*-tests and one-way analysis of covariance) and chi-square statistics were performed. The conditions for using this method (homogeneity of variance, normal distribution) were met.

The data were analysed using SPSS 20.0 for Windows. Means, standard deviations, and confidence intervals were calculated to describe the sample and illustrate differences in mean values. The chi-square test was used to test for differences in frequencies of specific characteristics of the preschools (e.g., size and location) and the children (e.g., sex, socioeconomic status, and migration background).

Socioeconomic status (SES) was assessed based on self-reported information from the parents concerning parental education/training level, professional status, and net income of all household members using the Winkler Index [38]. Children with a migration background were defined as those in which both parents have a migration background.

Variance analysis methods were used to test for differences in physical activity, social behaviour and QoL between preschools with systematic physical activity programmes and those with no physical activity programmes. The differences between physical activity levels by parental factors were also analysed by variance analysis. Sex, SES and migration background were tested as covariates. An alpha level of 0.05 was used to define statistical significance.

The aim of qualitative analysis of the observation logs created in the ethnographic study was to obtain differentiated insight into the specific physical activities performed and their social contextualization in the interaction forms and structure of activities offered by the day care centres. The qualitative observation logs were analysed on the basis of open and theoretical coding [39]. This included detailed analysis of selected data, particularly problematic and especially relevant passages of observation protocols. The coding was intended to break down the existing structure of the reports. The development of a category system made it possible to compare the data with each other and to contrast them with the quantitative research results. 

### 2.2. Integration of Quantitative and Qualitative Data

Finally, results from the quantitative and qualitative analyses were linked to illuminate different aspects of systematic physical activity promotion in preschool using the triangulation of different methods in conjunction with cross-validation and supplementation of the generated data [40]. The present study used a phase design [27] featuring the separate and successive use of both qualitative and quantitative research methods. The stage at which integration of the different methodologies occurred was during the interpretation of results. 

The combination of qualitative and quantitative research is often characterized by the results obtained. According to Kelle and Erzberger [28] results may by either: (1) convergent (*i.e.*, tend to agree), (2) complementary (*i.e.*, mutually supportive), or (3) divergent (*i.e.*, contradict each other). The triangulation of quantitative and qualitative data also has current validity in terms of the two-way analysis and justification of research results. In the present study, qualitative data were used to enable to a differentiation and deeper understanding of the quantitative study data.

## 3. Results

We interviewed 227 children and 193 parents at 21 preschools with systematic physical activity programmes (average 11 children/preschool), and 190 children and 170 parents at 25 preschools without physical activity programmes (average eight children/preschool). The average number of children interviewed at preschools without physical activity programmes was lower because the average non- physical activity facility was smaller than the average systematic physical activity preschool [23]. To compensate for this, a larger number of preschools without physical activity programmes were included in the survey. There was an approximately equal distribution of participants by sex, SES and migration background in the two preschool type groups. Overall, the number of participating parents (n = 363) was slightly lower at preschools without physical activity programmes (see Table 1).

**Table 1:** Descriptive characteristics of participants by preschool type.

Participants	Characteristics	Type I (Preschools with Systematic PAPs)		Type II (Preschools without PAPs)		Total	
**Children**		227	(54.4%)	190	(45.6%)	417	(100.0%)
**Sex**	Male	121	(53.3%)	97	(51.1%)	218	(52.3%)
	Female	106	(46.7%)	93	(48.9%)	199	(47.7%)
**SES**	Low	97	(50.3%)	76	(44.7%)	173	(47.7%)
	High	96	(49.7%)	94	(55.3%)	190	(52.3%)
**Migration background**	No	163	(84.5%)	146	(85.9%)	309	(85.1%)
	Yes	30	(15.5%)	24	(14.1%)	54	(14.9%)
**Parents**		193	(53.2%)	170	(46.8%)	363	(100.0%)

Notes: PAP: physical activity programme; SES: socioeconomic status.

Pedometer data from 113 children (61 from preschools with systematic physical activity programmes and 52 from preschools without physical activity programmes) were available for analysis. Data from the remaining seven children had to be excluded because the children either lost the pedometer or wore it for less than five days.

The results regarding the differences in children’s physical activity, QoL and social behaviour between preschools with systematic physical activity programmes and preschools without physical activity programmes as well as the differences in children’s physical activity according to parent-related factors are presented below. 

### 3.1. Physical Activity

First, we tested for differences in children’s physical activity levels (n = 113 children; 62 boys and 51 girls) during preschool hours between the preschools with systematic physical activity programmes and those without physical activity programmes. The mean number of steps during preschool hours (8 to 12 a.m.) did not differ significantly between the two preschool types; this held true for boys (*p* = 0.709, adjusted for SES) as well as girls (*p* = 0.887, adjusted for SES) (Table 2). 

**Table 2:** Differences in children’s physical activity, social behaviour and quality of life according to preschool type.

Health Resources	Characteristics		Mean ± SD				*p*
			Preschools with Systematic PAPs		Preschools without PAPs		
**Physical activity**	Steps/preschool hours (a.m.)	Girls	1,720.00	(721.00)	1,791.00	(608.00)	0.709
		Boys	2,180.00	(1,136.00)	2,221.00	(1,096.00)	0.887
	Steps/day after preschool	Girls	2,718.00	(1,760.00)	2,370.00	(1,232.00)	0.443
		Boys	3,085.00	(1,687.00)	3,567.00	(1,910.00)	0.343
	MVPA	Girls	167.00	(99.00)	163.00	(104.00)	0.827
		Boys	180.00	(113.00)	189.00	(113.00)	0.624
**Social behaviour**	Peer problems score	Girls	1.20	(0.35)	1.23	(0.35)	0.437
		Boys	1.25	(0.36)	1.13	(0.26)	0.023 *
	Prosocial score	Girls	2.74	(0.30)	2.69	(0.40)	0.311
		Boys	2.52	(0.45)	2.64	(0.40)	0.059
**Quality of life **	Self-reported		73.40	(12.60)	74.30	(13.60)	0.597
	Parentally reported		81.10	(7.80)	81.60	(7.60)	0.516

Notes: PA: physical activity; *p*: *p*-value associated with the one-way analysis of covariance (ANCOVA); MVPA: moderate-to-vigorous physical activity (MVPA) in minutes per week (parentally reported); PAP: physical activity programme; SD: standard deviation; * Statistically significant difference.

Second, we examined the extent to which the children’s after-preschool physical activity behaviours differed between preschools with systematic *versus* no physical activity programmes. The results showed no significant difference in the number of steps per day after preschool between the two preschool types (*p* = 0.981, adjusted for sex and SES). Likewise, the analysis conducted using parentally reported children’s MVPA (minutes/week) as the outcome variable showed no significant differences (*p* = 0.232) between the two preschool types (adjusted for sex and SES) (Table 2).

In contrast, our analysis of the qualitative observations in four preschools (two with systematic physical activity programmes and two without physical activity programmes) revealed differences between the physical activity of children in the two preschool types. For example, it was found that systematic physical activity promotion can lead to a more structured preschool day. The children at facilities with systematic physical activity programmes were involved in a variety of different, mostly guided activities with a specific curricular focus, such as the promotion of coordination or motor skills. It is particularly noteworthy that, based on the observation logs, the children (especially boys) at a large preschool with a comprehensive physical activity programme were more physically active during structured physical activities initiated by teachers as well as during self-initiated physical activities such as ball games or “catch” than those at smaller preschools without physical activity programmes.

In addition, we found that preschools with systematic physical activity programmes offered a larger range of physical activities, both indoor and outdoor, than preschools without systematic physical activity programmes.

In agreement with the quantitative analysis, the qualitative observation study showed that boys are more physically active than girls. During breaks, girls tended to stay in the classrooms and engage in activities such as arts, crafts and painting, while the boys tended to take over the physical activity facilities and often appeared to crowd girls out of the physical activity areas.

### 3.2. Social Behaviour

Boys in the preschools with systematic physical activity programmes had significantly higher peer problems scores than those in the preschools without physical activity programmes (*p* = 0.023, adjusted for SES). Only 4% of boys in the preschools without physical activity programmes had “at-least-borderline” behaviour compared to 10% of those in the preschools with systematic physical activity programmes. Girls showed no significant differences between preschool types (*p* = 0.681, adjusted for SES). 12% of the girls in preschools with systematic physical activity programmes *versus* 13% of the girls in preschools without physical activity programmes demonstrated “at-least-borderline” peer problems. 

The differences in prosocial scale scores between preschools with systematic physical activity programmes and those without physical activity programmes were not significant for boys (*p* = 0.059, adjusted for SES) or girls (*p* = 0.311, adjusted for SES) (Table 2). 23% of boys and 4% of girls in preschools with systematic physical activity programmes had “at least borderline” behaviour compared to 16% of boys and 11% of girls in preschools without physical activity programmes.

In contrast, analysis of the qualitative observational study data showed that children in preschools with systematic physical activity programmes exhibited marked independence and social skills, which resulted in a relatively harmonious atmosphere and low noise levels in this group. The children communicated the prevailing rules of their preschool, especially those taught in the context of physical activity exercises, and often resolved conflicts among themselves. Aggressive scuffling was rarely observed among the children in this group. On the other hand, it was conspicuous that the children in preschools without physical activity programmes communicated the preschools’ rules much less frequently, and that they found these rules to be non-transparent in some cases. Moreover, children in all-day groups in the two preschools without physical activity programmes seemed to require much attention. Peer interaction was often marked by conflict, particularly among the boys, and noise levels were relatively high. These children were less able to engage in independent activities, seemed restless, and tended to resort to physical conflict resolution strategies.

### 3.3. Quality of Life

There were no significant differences in self-reported QoL between preschools with systematic physical activity programmes and those without physical activity programmes (*p* = 0.597, adjusted for sex and SES). Likewise, parentally reported children’s QoL values did not differ significantly between the two preschool types (*p* = 0.516) (Table 2).

### 3.4. Parent-related Factors

**Figure 1:**
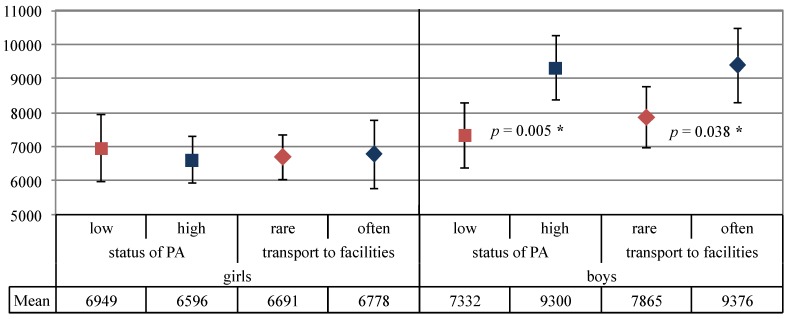
Children’s (n = 113) number of steps per day (mean ± 95% CI) stratified by parental attitudes (towards physical activity), parental support (transport to facilities) and sex.

Note: * Statistically significant difference.

The following parent-related factors were examined to determine the extent to which children’s physical activity differs by parental socialization behaviour: (a) parental support behaviour (taking the child to facilities), (b) parental attitudes towards physical activity (role of physical activity in the family), and (c) parental role model behaviour (joint parent-child physical activity). The children’s mean number of steps per day was defined as the outcome variable. Boys with frequent parental support (*i.e.*, those whose parents took them to sports facilities at least 3 to 5 times/week) had a significantly (*p* = 0.038) higher number of steps than boys with less frequent parental support. This did not apply to girls (*p* = 0.886). Boys whose families placed high emphasis on physical activity had a significantly (*p* = 0.005) higher number of steps than boys whose families placed low emphasis on physical activity. Again, girls showed no significant differences in this variable (*p* = 0.556) (see Figure 1).

There was no significant difference in children’s number of steps per day regarding parental role model behaviour; this applied to both boys (*p* = 0.528) and girls (*p* = 0.857). The parentally reported children’s MVPA (minutes/week) differed significantly by all three parent-related factors. This result was also observed in girls in all three factors except “Parental attitudes” (see Table 3).

Our analyses of the qualitative observational study confirm that there is an association between parental behaviour and children’s physical activity. Interestingly, analysis of the ethnographic interviews revealed that parents whose children had especially good motor skills encouraged the children’s physical activity more frequently. 

**Table 3:** Parent-related factors and parentally reported children’s MVPA.

Parent-related Factors	Characteristics		Mean ± SD		*p*	η_p_^2^
**Parental support**	Girls	Often	190.00	(94.4)	0.014 *	0.041
		Seldom	147.67	(91.7)		
	Boys	Often	224.44	(123.7)	0.001 *	0.082
		Seldom	158.28	(98.5)		
**Parent-child activity**	Girls	Often	197.66	(118.8)	0.006 *	0.053
		Seldom	149.84	(81.6)		
	Boys	Often	218.77	(125.2)	0.005 *	0.053
		Seldom	164.27	(102.3)		
**Role of PA in the family**	Girls	High	170.07	(99.0)	0.286	0.008
		Low	149.46	(107.0)		
	Boys	High	198.73	(103.0)	0.006 *	0.052
		Low	145.61	(111.0)		

Notes: Mean: mean moderate-to-vigorous physical activity (MVPA) in minutes per week; PA: physical activity; *p*: *p*-value associated with the one-way analysis of covariance (ANCOVA); SD: standard deviation; ηp^2^: partial eta squared associated with the one-way ANCOVA; * Statistically significant difference.

The following representative scene documented in an observation log describes how a father promoted the physical activity of his son, who had very good motor skills:

*“I saw Marco’s father and interrupted my conversation with the teacher to ask for his consent to participate in the observational study. Beforehand, I had watched the father and son running down the corridor to the group room. Mr. T. called out, “Let’s see who gets there first!” Marco, who was walking ahead, laughed and ran away from his father, who chased after him. The two of them came to a halt in front of the group room and said their goodbyes”.*

(*observation at a preschool with a systematic physical activity programme, log entry on 14 November 2011*)

Hence, there is evidence suggesting unconscious mechanisms leading to gender-specific promotion of physical activity exist, and can be conveyed even though they contradict the actual educational intent. 

## 4. Discussion

The present article describes the results of our analysis of differences in children’s health resources by the presence *versus* absence of physical activity programmes in preschools. Similarly, we assessed the differences in children’s physical activity by parental behaviour. Physical activity is a complex multidimensional behaviour that is determined by a variety of different biological, psychological, socio-cultural and environmental factors [14]. According to the social ecological model, family and teachers are the two main factors that influence the physical activity behaviour of preschoolers [20]. That is why this study targets these settings.

The use of a mixed methods triangulation approach, including a combination of standardised survey instruments and focused ethnography, provided comprehensive insight into the nature, scale and routine practical implementation of (systematic) physical activity programmes in preschools. Data triangulation allowed us to distinguish different aspects of the associations between systematic physical activity promotion and children’s health resources and provided data of higher validity. The present study was the first of this design conducted in Germany. 

The recruitment of parents and children was a challenge. Since the participants were recruited by the preschools, the researchers had no personal contact with the parents. Thus, it is possible that the parents’ motivation to participate was influenced by the teachers’ attitudes towards the study. This could have a positive effect on participation if the parents have trust in the teachers. Conversely, negative attitudes of teachers can have a deterrent effect on parental participation. It is also possible that the majority of parents who participated in the study place a high value on physical activity. Consequently, selection bias of the participants cannot be excluded. 

So far, only a few standardised instruments are available for interviewing the children under ten years of age [41]. The instruments used in the present study proved to be well-suited for the interviews with children. 

The use of objective measurement tools such as pedometers reduces the subjectivity of survey methods and the likelihood of response bias. Pedometers are small, convenient, non-intrusive and cost-efficient devices that are regarded as being very accurate step counters [42,43]. As a caveat, it must be noted that pedometers only provide a general measure of physical activity and do not provide information about the site and type of physical activity (e.g., walking *versus* playing), and do not record upper body or horizontal movement. Moreover, pedometers are not accurate at estimating distances or energy expenditure. Nonetheless, pedometers have proven to be a good objective tool for measuring robust physical activity levels in this age range [43]. 

Another limitation is the use of a cross-sectional design, which prevents us from drawing conclusions about causal relationships and changes.

The qualitative observational study also has a potential for bias as the subjectivity of the observer is a key instrument of understanding the observation process. That which is observed always depends on the individual observer, who brings his or her specific perception, thinking and action strategies to the observation field [44]. However, the use of different methodological approaches resulted in mutual supplementation and testing of the generated data, which is indeed useful [40]. 

Linkage of the quantitative and qualitative survey data revealed divergent findings in several areas. Contradictions between the results obtained by quantitative and qualitative methods are not uncommon; this can contribute to the revision and modification of theoretical assumptions and prompt the development of new theoretical concepts [45]. 

The present study used a combination of qualitative and quantitative methods, in particular, to generate research results that are mutually complementary. By contrasting the quantitative and qualitative data, we obtained evidence of facts and contexts that could not have been gained using the quantitative data alone. Thus, the qualitative data enabled a differentiation and deeper understanding of the quantitative data. The results of qualitative and quantitative studies may be mutually convergent, complementary or contradictory, and each of these possibilities can be fruitful for the research process [28]. In the present study, both similarities and differences in the quantitative and qualitative results were detected. 

### 4.1. Preschool-related Factors

#### 4.1.1. Differences in Physical Activity

The present study examined the extent to which the children’s physical activity and social behaviour differs between preschools with systematic physical activity programmes and those without physical activity programmes. Contrast analysis of the quantitative and qualitative data yielded divergent results. No differences in children’s physical activity between the preschool types could be identified based on the quantitative data. The results showed that there was no significant difference in the number of steps/day during preschool hours between the two preschool types. We also tested for differences in the children’s physical activity behaviour between the two preschool types during after-preschool hours. Neither the pedometer data nor the parentally reported data showed any significant differences in the children’s after-preschool physical activity behaviour between the two preschool types. 

The qualitative observational study, on the other hand, showed that children (especially boys) in preschools with systematic physical activity programmes participate in both structured and self-initiated physical activities during preschool hours more often than those in preschools without physical activity programmes. Consequently, it is assumed that boys benefit more from preschool physical activity programmes than girls. Based on the qualitative data, it can be concluded that preschoolers have higher activity levels under certain conditions.

The observation logs provided evidence of higher structural and content quality of the physical activity programmes at preschools with systematic physical activity programmes. For example, preschools with systematic physical activity programmes provided a much more differentiated range of physical activities in which specific physical and motor skills were promoted in a targeted manner. Therefore, the type and intensity of children’s physical activity differed between the two preschool types. In addition, the preschools with systematic physical activity programmes offered a greater number and range of indoor and outdoor physical activities than those without physical activity programmes.

The fact that our quantitative data analysis revealed no significant differences in the children’s number of steps between the two preschool types may be explained by the fact that children of this age have a natural tendency to be physically active. According to the activity stat theory, young children in particular have an individual physical activity ‘controller’ that leads to compensatory increase in physical activity after mandatory periods of inactivity [46]. Consequently, children’s mean daily activity levels were found to remain around the same “set point”, even if they attended preschools with exercise programmes designed to increase their activity levels [46]. Children in preschools with systematic physical activity programmes as well as those in preschools without physical activity programmes have ample opportunity to satisfy their basic need for physical activity. It must be noted that pedometers do not allow a differentiated analysis of the type and intensity of physical activity. However, studies have demonstrated that structured physical activity programmes and a physical activity-friendly preschool environment increase the physical activity levels and improve the body composition of young children [18,47,48]. 

Triangulation of our quantitative and qualitative data provided a differentiated picture of the associations between children’s physical activity behaviour and preschool physical activity programmes. The qualitative observations gained from participation in the field provided additional insights that could not have been revealed by the quantitative data. Further studies are needed to determine the impact of structured preschool physical activity programmes on children’s physical activity in more detail [49]. 

#### 4.1.2. Differences in Social Behaviour and Quality of Life

The quantitative and qualitative analyses of the children’s social behaviour yielded divergent results. While the quantitative data showed that boys in preschools with systematic physical activity programmes have significantly more peer relationship problems than those in preschools without physical activity programmes, the observation study suggested a different picture. The observations showed that peer interactions of children (especially boys) at preschools without physical activity programmes were more often characterized by conflict than those of children in preschools with systematic physical activity programmes. 

It can be assumed that the structured pedagogic concepts of preschools with systematic physical activity programmes, in combination with their structured physical activity promotion measures and the promotion of children’s self-reliance, can have a positive impact on children’s social behaviour. This positively affects children’s self-efficacy and social skills as well as teacher-child interactions. Physical activity also promotes the children’s communication of rules and self-confidence. This effect could not be determined based on the quantitative data because the instruments used in the study do not capture everyday life at the preschools through participation in the field. 

A possible explanation for the quantitative results regarding peer interaction is that problematic social behaviour might be more likely be noticed and negatively sanctioned at preschools with systematic physical activity programmes, where cooperative behaviour is required and encouraged, especially in the context of physical activity offerings. 

Regarding the children’s QoL, no significant differences between the two preschool types could be detected based on the quantitative data. Studies have shown that physical activity during childhood can contribute to a better QoL [32]. However, the results are generally inconsistent. 

### 4.2. Parent-related Factors

According to the social cognitive theory of Bandura [25], the parent-child relationship is a central correlate of children’s physical activity, and the concept of observational learning is a core aspect of this theory. The observation of role models such as parents can result not only in behavioural changes but also in cognitive and emotional changes.

In view of the influence of parent-related behaviour (parental support, parental attitudes towards physical activity and parental role model behaviour) on children’s physical activity, the qualitative and quantitative findings are complementary. The quantitative and qualitative results show that the children’s physical activity behaviour differs according to parental socialization behaviour. Although there are a number of other socialization agents besides parents (peers, preschool, *etc*.), the socialization of health behaviour occurs within the family. Physical activity behaviour, especially that of young children, is thus significantly influenced by parental convictions, attitudes and behaviours [6,14,23]. This was also indicated by the present results. 

Our results also showed that supportive parental behaviour seems to be more important for the physical activity behaviour of boys than girls. The qualitative results also suggest that parents act supportively, especially when their children enjoy physical activity and demonstrate good motor skills. This finding leads to the hypothesis that a high level of parental support promotes children’s physical activity [23] and, conversely, that children with high physical activity levels receive greater parental support. The direction of this association should be examined in detail in longitudinal studies. Moreover, the gender-specific needs of girls and boys regarding physical activity promotion should be given greater consideration in the future.

## 5. Conclusions

Combined analysis of our quantitative and qualitative data made it possible to elucidate different perspectives and thus gain a comprehensive, holistic picture of systematic physical activity promotion in preschools. Qualitative and quantitative methods often provide information intended to explain surprising and/or incomprehensible results obtained using other methodologies or to correct the misinterpretation of findings. To date, hardly any uniform conceptual foundations exist for mixed methods data combination and analysis [28]. 

A limitation of the study is the use of a cross-sectional design. Moreover, selection bias of the participants (e.g., preschools and parents) cannot be excluded. Objective measurement tools such as pedometers reduce the subjectivity of survey methods and the likelihood of response bias. However, pedometers only provide a general measure of physical activity and do not provide information about the type and intensity of physical activity.

This study confirms the assumption that parental behaviour is one of the most important factors influencing children’s physical activity and that parental influence is probably greater than preschool influence. Future research should examine the question of how and in which form parents and the home environment can be more strongly included in physical activity promotion programmes for preschool children.

Like family, preschool is also a central pillar of child care and education and thus an ideal setting for physical activity promotion and its associated health benefits [6]. Therefore, the potentials of preschools to promote child health resources should be used more effectively. Physical activity promotion should be an integral part of preschool education policy, and programmes to promote children’s physical activity should be permanently implemented in the daily routine of preschools. 

Preschoolers show a high degree of responsiveness to changes in the day care or home environment. It can be assumed that interventions that include teachers and parents may produce strong effects such as changes in healthy active living habits in preschoolers [6]. However, there are many indications that the only way to increase and enhance the effectiveness of such measures is by cooperating with parents. Moreover, future physical activity promotion interventions should consider the gender-specific needs of girls and boys to a greater extent. 

More studies using differentiated methods, such as a mixed methods and longitudinal design, are needed to assess the effectiveness of physical activity promotion interventions in preschools.
